# The Medicinal Species of the Lycium Genus (Goji Berries) in East Asia: A Review of Its Effect on Cell Signal Transduction Pathways

**DOI:** 10.3390/plants13111531

**Published:** 2024-05-31

**Authors:** Chenyu Jiang, Ziyu Chen, Weilin Liao, Ren Zhang, Geer Chen, Lijuan Ma, Haijie Yu

**Affiliations:** Dr. Neher’s Biophysics Laboratory for Innovative Drug Discovery, State Key Laboratory of Quality Research in Chinese Medicine, Macau University of Science and Technology, Macau 999078, China; jiangchenyu577@gmail.com (C.J.); ziyuc643@gmail.com (Z.C.); idiotlinlee@gmail.com (W.L.); zr111594@gmail.com (R.Z.); chengeer999@gmail.com (G.C.)

**Keywords:** Lycium genus, goji berries, signal pathway, gut microbiota, pharmacological activity

## Abstract

Natural plants contain numerous chemical compounds that are beneficial to human health. The berries from the Lycium genus are widely consumed and are highly nutritious. Moreover, their chemical constituents have attracted attention for their health-promoting properties. In East Asia, there are three varieties of the Lycium genus (*Lycium barbarum* L., *Lycium chinense Miller*, and *L. ruthenicum Murray*) that possess medicinal value and are commonly used for treating chronic diseases and improving metabolic disorders. These varieties are locally referred to as “red Goji berries” or “black Goji berries” due to their distinct colors, and they differ in their chemical compositions, primarily in terms of carotenoid and anthocyanin content. The pharmacological functions of these berries include anti-aging, antioxidant, anti-inflammatory, and anti-exercise fatigue effects. This review aims to analyze previous and recent studies on the active ingredients and pharmacological activities of these Lycium varieties, elucidating their signaling pathways and assessing their impact on the gut microbiota. Furthermore, the potential prospects for using these active ingredients in the treatment of COVID-19 are evaluated. This review explores the potential targets of these Lycium varieties in the treatment of relevant diseases, highlighting their potential value in drug development.

## 1. Introduction

**Figure 1:**
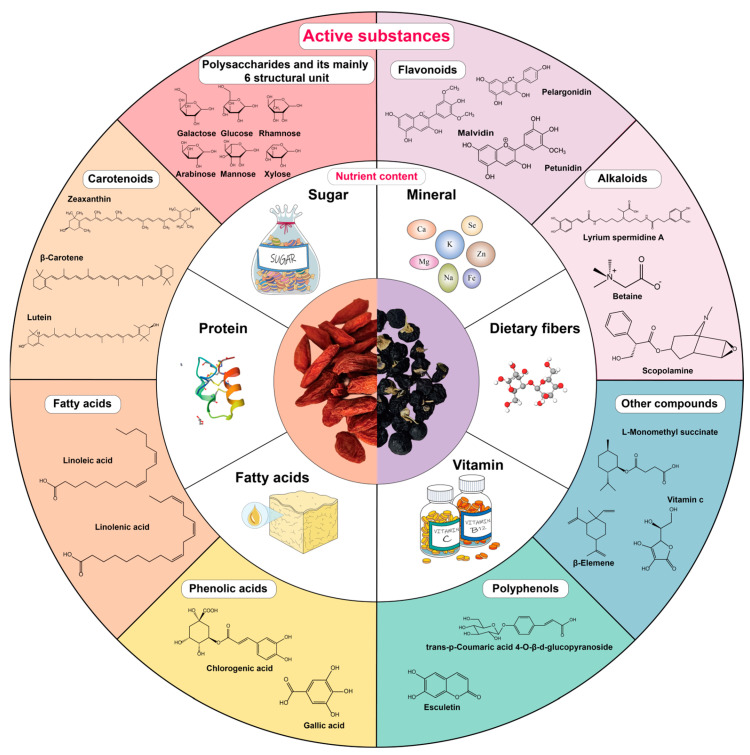
Chemical composition of red-fruited goji berry (LB, LCM) and black-fruited goji berry (LRM). These plentiful chemical compositions contribute to the distinctive properties and potential health benefits associated with red-fruited and black-fruited goji berries. The items in the white circle represent the nutritional components of goji berries, and the items in the colored circles represent the active ingredients in goji berries.

For thousands of years, medicinal plants containing natural active ingredients have been utilized in the treatment of various diseases and in promoting good health. The fruit of the genus Lycium plant serves as a remarkable example [1]. The Lycium genus is a group of plants in the family Solanaceae. Yao and his colleagues identified and named a total of ninety-seven species and six variants of Lycium plants found worldwide [2]. In East Asia, three primary medicinal species are found in the genus Lycium, namely *Lycium barbarum* L. (LB), *Lycium chinense Miller* (LCM), and *Lycium ruthenicum Murray* (LRM), which are commonly known as “Goji berry” in general terms. Specifically, due to their different colors, LB and LCM are often referred to as “red Goji berries”, while LRM is commonly known as “black Goji berries”. For the sake of simplicity, these three species will be collectively referred to as “goji berries” in this article. Additionally, native species can also be found in Europe, the Americas, and Africa [3,4]. Goji berries have been used for centuries in East Asia as both a natural medicine and health food. They are believed to have several key functions, including strengthening bones and muscles, promoting healthy movement, treating eye injuries, maintaining reproductive function, and potentially extending lifespans [5,6,7]. Although they contain a rich array of nutrients, including carbohydrates, vitamins, amino acids, inorganic salt, high-molecular polysaccharides, enzymes, and other biologically active ingredients, there are differences not only in the color of the fruit but also in their composition among the different species of goji berries [8,9,10,11]. LB and LCM are characterized by their red fruits and have higher levels of carotenoids and related components. On the other hand, LRM has black fruits and contains more anthocyanins, tannins, and phenolics [11], which may potentially lead to stronger antioxidant effects. The main components of these berries include polysaccharides (like Lycium barbarum polysaccharides (LBPs), generally consisting of six monosaccharides, including galactose, glucose, rhamnose, arabinose, mannose, and xylose) [12,13], betaine, and other compounds [14,15]. Red-fruited goji berries are rich in carotenoid components like Zeaxanthin, β-Carotene, and Lutein, while black-fruited goji berries contain higher levels of anthocyanin-like components and unique components such as lyrium spermidine A (Figure 1) [16]. Goji berries have various applications in society, such as being used as a food additive, health food, and natural medicine [17]. Their wide range of uses has contributed to their relatively high level of social acceptance.

Many human diseases are associated with various cell signaling pathways, which serve as important targets for therapeutic interventions. Understanding the signaling pathways involved in disease can lead to the discovery of effective therapeutic agents. In this paper, a recent review is presented on the active ingredients found in goji berries and the signaling pathways they impact. These pathways include the nuclear factor kappa-light-chain-enhancer of activated B cells (NF-κB), Fas cell surface death receptor and factor-related apoptosis ligand (Fas/Fasl), phosphoinositide 3-kinases and protein kinase B (PI3K-AKT), sirtuins (SIRTs), p38 mitogen-activated protein kinase (p38 MAPK), nuclear factor erythroid 2-related factor 2 and heme oxygenase 1 (NRF2/HO-1), N-methyl-D-aspartate receptor (NMDA) receptor-related pathway, and others. The paper also briefly explains the effects of goji berry-derived active ingredients on gut microbiota and their possible prospects for the treatment of COVID-19. The objective of this paper is to provide valuable insights and references for identifying disease targets and discovering drugs from natural plant components, with the ultimate goal of contributing to achieving precision medicine.

## 2. Review Strategy

The scientific names of the selected Lycium plants were confirmed on the “Plants of the World Online” database (http://www.plantsoftheworldonline.org/ (accessed on 22 February 2024)). And the literature search was carried out using the most common medical, biological, and chemical databases (such as Scopus, PubMed, and Web of Science). The keywords included a combination of the name of the selected Lycium plants and the relevant bioactivity and signal pathway. In addition, “Gut Microbiota” and “COVID-19” were used as secondary keywords. We selected and assessed review articles and original research articles published in English up to March 2024. All the search outcomes were carefully looked at to select only the studies that were relevant to the scope of this review.

## 3. Goji Berries’ Active Ingredients with Signal Pathways

Goji berries contain a variety of active ingredients that can impact human health (Table 1). The following are the main signaling pathways that they can affect.

### 3.1. NF-κB Signal Transduction Pathway

NF-κB is a class of nuclear transcription factors that play a significant role in regulating the expression of genes. These genes are involved in various important biological processes, such as cell proliferation, apoptosis, inflammation, tumorigenesis, and viral replication [31,32,33,34]. The activation of NF-κB is triggered by a wide range of physiological and pathological stimuli. It utilizes a variety of receptors, including pattern–recognition receptors (PRRs), T-cell receptors (TCRs), B-cell receptors (BCRs), proinflammatory cytokine receptors, and other receptors [35,36], to receive versatile biological signals. These signals can come from growth factors, inflammatory factors, chemokines, and even environmental stimuli such as ultraviolet radiation and chemical toxicity [37,38,39].

Goji berries contain active ingredients that can regulate the NF-κB signaling pathway, leading to a variety of biological effects. Lycium barbarum polysaccharides (LBPs) activate the NF-κB pathway through Toll-like receptors (TLRs), promoting the activation and maturation of antigen-presenting cells. For example, the engineered liposomes of LBPs are designed to enhance immune system vitality by targeting dendritic cells (DCs) and promoting immune response [40]. Based on this principle, LBPs have the potential to be used as an immune adjuvant [41]. Additionally, LBPs have been found to inhibit aberrant NF-κB activation induced by carbon tetrachloride (CCl_4_); reduce the expression of inflammation-related factors such as tumor necrosis factor-alpha (TNF-α), inducible nitric oxide synthase (iNOS), interleukin 1 beta (IL-1β), and cyclooxygenase-2 (COX-2); and alleviate acute liver injury and hepatic fibrosis caused by CCl_4_ in mice and rats [42,43].

**Figure 2:**
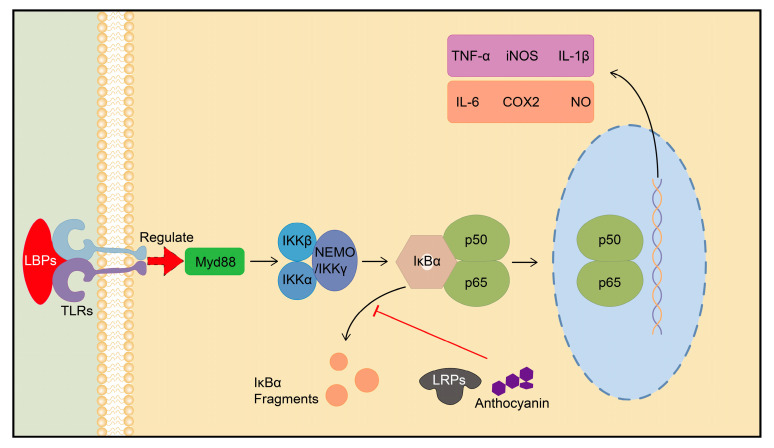
Regulation of NF-κB signaling pathway by active ingredients of goji berries. Myd88: Myeloid differentiation factor 88, IKK: IkappaB kinase.

Polysaccharides obtained from *Lycium ruthenicum Murray* (LRPs) can also regulate the Toll-like receptor 4 (TLR4)/NF-κB signaling pathway. LRPs inhibit this pathway by preventing the degradation of the inhibitor of nuclear factor kappa B (IκBα) and blocking the phosphorylation of transcription factor p65 (p65), thereby reducing LPS-induced inflammatory responses in mouse macrophages [44]. LRPs have also been found to promote cancer cell apoptosis and exhibit in vivo anti-tumor effects in the BxPC-3 pancreatic cancer cell line. These effects are achieved by inhibiting the p38 MAPK/NF-κB transcriptional co-activation system while not affecting the function of NF-κB in normal cells or organisms [45,46]. Furthermore, anthocyanins in LRM can affect the NF-κB signaling pathway and inhibit D-galactose-induced NF-κB overexpression and neuroinflammation This leads to an improvement in cognitive function and memory ability in adult rats [47,48]. LRPs can also reduce the levels of signaling molecules associated with the NF-κB pathway, such as TLR4, transforming growth factor beta 1 (TGF-β1), and interleukin-6 (IL-6), ameliorating high-fat diet (HFD)-induced liver inflammation, oxidative stress, and insulin antagonism (Figure 2) [49].

### 3.2. Fas/Fasl Signal Transduction Pathway

Fas and Fas ligand (FasL) are two molecules that regulate cell death by activating apoptosis-related molecular signals through Fas-associated death domain protein (FADD)-mediated caspase-8 activation. Fas/Fasl-mediated apoptosis is a crucial mechanism for maintaining homeostasis and has significant implications in the biological aging process of the body, being particularly associated with the aging of the reproductive system [50,51]. It is also correlated with the pathology of chronic diseases such as diabetes, neurodegenerative diseases, and cancers [52,53].

**Figure 3:**
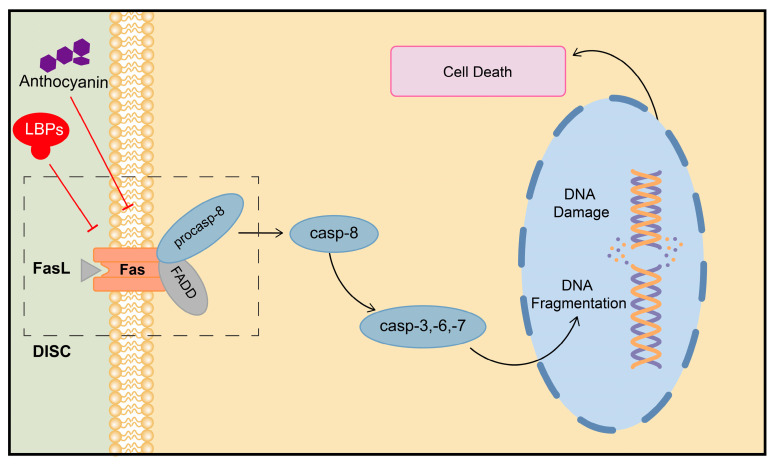
Regulation of Fas/Fasl signaling pathway by active ingredients in goji berries. DISC: death-inducing signaling complex.

Recent studies have indicated that goji berries can provide benefits in terms of anti-aging and fertility protection by modulating the activity of the Fas/Fasl signaling pathway. In a rat model of oxidative stress-mediated aging-related liver injury induced by D-galactose, anthocyanins in LRM (LRA) effectively mitigated hepatocellular injury, necrosis, and the inflammatory response by downregulating the mRNA expression level of Fas/Fasl. LRA also reduced the serum activities of aspartate transaminase (AST) and alanine aminotransferase (ALT), indicating the protective effect of LRA [54]. Furthermore, germ cells are highly susceptible to environmental, physical, and chemical factors, which can lead to the abnormal activation of Fas/Fasl. This aberrant process can induce the premature apoptosis of germ cells and consequently contribute to infertility issues [55]. Research has shown that LBPs can enhance the proliferation and improve the function of amice TM4Sertoli cells. Additionally, LBPs can significantly inhibit the activation of Fas/Fasl induced by 2,4-Dichlorophenoxyacetic acid (2,4-D) in rats. This inhibition leads to a reduction in the levels of caspase-8, caspase-3, and other proteins in the related signaling pathway, ultimately ameliorating germ cell apoptosis and mitigating testicular tissue damage [56]. (Figure 3) These findings suggest that goji berries could be utilized as a daily supplement for fertility protection.

### 3.3. PI3K-AKT Signal Transduction Pathway

The PI3K family of phosphatidylinositol kinase is responsible for regulating various cellular processes. Through its downstream mediators, such as AKT, PI3K plays a crucial role in controlling biological functions, including angiogenesis, lipid metabolism, and the maintenance of the normal cell cycle. The dysregulation of this pathway is implicated in cancer development and is closely associated with metabolic diseases like diabetes and obesity [57,58,59,60]. AKT regulates multiple important cellular signaling pathways, including the AKT–Forkhead box protein O (FOXO), AKT–the mammalian target of rapamycin (mTOR), and AKT–glycogen synthase kinase 3 (GSK3) pathways. Its function can also be regulated through 3-phosphoinositide-dependent protein kinase (PDK) and PH domain and Leucine-rich repeat protein phosphatases (PHLPPs). Additionally, phosphatase and tensin homolog (PTEN) negatively regulate the above signaling pathways by modifying the lipid composition of the cell membrane [59,61,62,63].

The active components of goji berries affect the signaling pathways mentioned above. For instance, in a nonalcoholic steatohepatitis (NASH) rat model induced by a high-fat diet, the inhibition of PI3K leads to FOXO1 activation, which plays a crucial role in hepatic stellate cell (HSC)-induced hepatic fibrosis. LBPs can reverse this process and have antifibrotic effects in the liver [64]. Likewise, betaine in LCM activated AKT signaling and inhibited FOXO1-induced NOD-like receptor protein 3 (NLRP3) inflammasomes [65]. In terms of anti-tumor activity, LBPs and Lycium barbarum glycopeptide (LbGp) have been demonstrated to inhibit the PI3K-AKT-mTOR pathway. This leads to apoptosis in infantile hemangioma endothelial cells (HemECs) [66] and blocks lipid synthesis in glioblastoma [67], respectively. Additionally, the potential anti-tumor properties of anthocyanins and polysaccharides from LRM should also be considered. They can interfere with the PI3K-AKT and Janus kinase 2 (JAK2) and activator of transcription 3 (STAT3) signaling pathways, resulting in apoptosis in the LoVo and HepG2 tumor cells [68]. Meanwhile, anthocyanin monomer Pt3G in LRM has been shown to increase the expression of PTEN in prostate cancer DU-145 cells, leading to the inhibition of the PI3K-AKT-mTOR pathway and inducing cell apoptosis [69]. In the area of body functional protection, LBPs were found to activate PI3K-AKT-mTOR signaling and reduce the expression level of Beclin-1, thereby inhibiting reproductive dysfunction triggered by aberrant autophagy in the testes of diabetic mice [70,71]. When combined with dodder *Cuscuta chinensis Lam*, LBPs could activate PI3K-AKT-mTOR signaling and downregulate the ratio of BCL2-Associated X (Bax)/B-cell lymphoma-2 (Bcl-2). This leads to a reduction in apoptosis and improved sperm counts and sperm viability [72]. In addition, LBPs upregulate PI3K-AKT phosphorylation, inhibit GSK-3β activity, and protect brain neuron cells from ischemia–reperfusion injury (IRI) in stroke mice [73]. Additionally, LBPs show antioxidant effects on rat aortic endothelial cells which are associated with the downregulation of reactive oxygen species (ROS) through the PI3K-Akt-mTOR signaling pathway [74].

**Figure 4:**
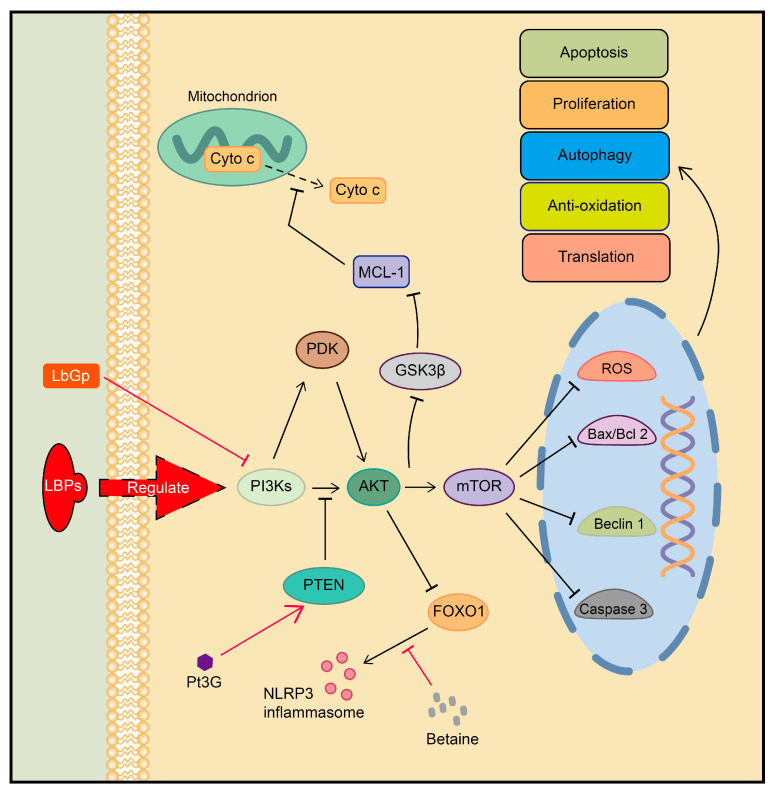
Regulation of PI3K-Akt signaling pathway by active ingredients in goji berries. MCL-1: Myeloid cell leukemia-1, Cyto c: Cytochrome c.

In summary, goji berries play an important role in exerting anti-inflammatory, antioxidant, anti-aging, and anti-tumor activities; reproductive protection; and neuroprotection via the PI3K-Akt pathway. In research for applications, a fibronectin hydrogel was prepared using Lycium barbarum oligosaccharide (LBO) with nasal mucosa-derived mesenchymal stem cells. This hydrogel can modify the microenvironment through cell paracrine effects, specially influencing the microglia PI3K-AKT-mTOR pathway and promoting the repair of spinal cord injuries in rats. This innovative application demonstrates the potential of utilizing the active ingredients found in goji berries [75] (Figure 4).

### 3.4. SIRT Signal Transduction Pathway

Sirtuins (SIRTs) are a family of proteins with mono-ADP-ribosyltransferase or deacylase activity. There are seven family members (SIRT1-7), each with distinct subcellular localizations and substrate specificities [76]. They are capable of sensing the level of nicotinamide adenine dinucleotide (NAD+) in the cell, which correlates with cellular energetic states, allowing them to adaptively regulate cellular functions [77]. The SIRT protein family plays a significant role in regulating the cell cycle, cellular metabolism, and aging. Moreover, it has been implicated in various disorders, such as cardiovascular disease, cancer, metabolic liver disease, and endocrine disorders [78,79,80,81,82].

The active ingredients in goji berries have been found to modulate SIRT1-related signaling pathways. For example, LBPs are able to activate the SIRT1/LKB1/AMPK pathway by increasing the NAD+/NADH ratio to increase acetyl coenzyme A carboxylase (ACC) phosphorylation and adipose triglyceride lipase (ATGL) expression, resulting in lipolysis activation and fatty acid synthase (FAS) reduction. Consequently, this modulation helps prevent and ameliorate nonalcoholic fatty liver disease (NAFLD) induced by a high-fat diet [83]. The betaine in LCM also activates SIRT1, leading to an increased expression of peroxisome proliferator-activated receptor gamma coactivator α (PGC1α), nuclear respiratory factor (NRF-1), and mitochondrial transcription factor A (TFAM). This activation promotes myocyte glucose uptake, promotes mitochondrial biosynthesis, and enhances cellular energy metabolism, increasing muscle strength and mitigating muscle dysfunction [84].

**Figure 5:**
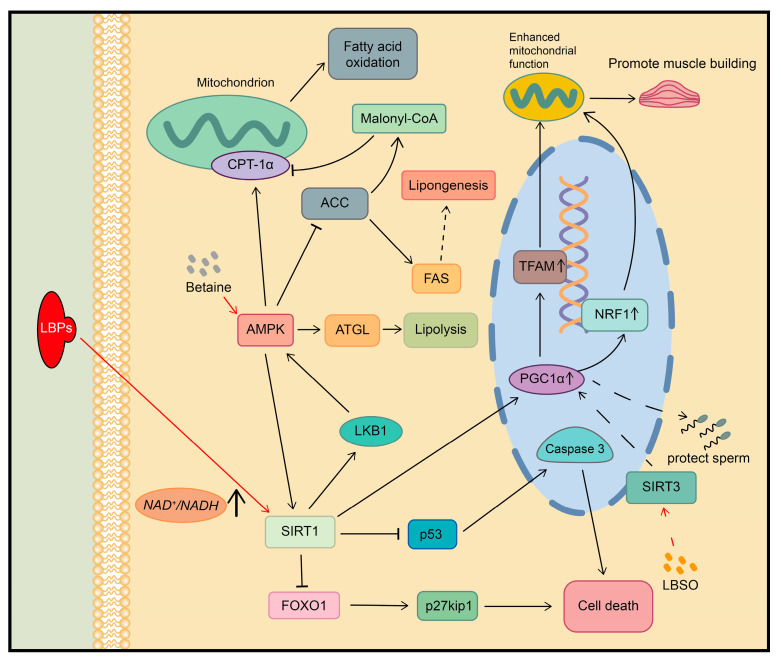
Regulation of SIRT signaling pathway by active ingredients in goji berries. CPT-1α: Carnitine palmitoyltransferase 1A.

LBPs were observed to have a protective effect in a rat model of diabetes-induced cataracts. This was attributed to the upregulation of SIRT1 expression and the downregulation of p53 and FOXO1 in lens tissue. These changes led to a reduction in caspase-3 and a decrease in cyclin-dependent kinase inhibitor 1B (p27kip1), ultimately protecting the lens tissue from cell death and delaying the development of diabetic cataracts [85]. LRM extracts significantly increase the lifespan of *Caenorhabditis elegans* nematodes by the activation of the Sir-2.1 protein, which shares structural similarity with the Sirtuins proteins [86]. Furthermore, a flavonoid glucoside found in LRM was identified to directly regulate the activity of the SIRT1 protein [87]. These findings highlight the potential of using LRM in targeted drug research for SIRT-related signaling pathways. In a D-galactose-induced rat model of reproductive aging, *Lycium barbarum* L. seed oil (LBSO) was found to improve mitochondrial function and reduce oxidative damage in the testes via the SIRT3/AMPK/PGC1α pathway [30]. In females, LBPs activate the Sirt1/AMPK-related signaling pathway, leading to improved ovarian autophagy. This can significantly protect healthy follicles, help maintain normal hormone levels, and effectively ameliorate D-galactose-induced premature ovarian failure (POF) in mice [88] (Figure 5).

### 3.5. p38 MAPK Signal Transduction Pathway

The mitogen-activated protein kinase (MAPK) cascade is a crucial mechanism for the cellular response to external signals. It involves a series of activated kinases, including MAPK kinase kinase (MAPKKK), MAPK kinase (MAPKK), and MAP kinase (MAPK), which deliver messages to downstream functional pathways [89,90]. Among them, the p38 MAPK subgroup is particularly important, as it responds to neurotransmitters, hormones, and environmental stimuli, influencing the cell cycle and cell fate. Furthermore, it plays a significant role in immune activation and inflammation. Various diseases, such as tumors, autoimmune diseases, pathological pain, and neurodegenerative diseases are associated with such signal pathways [91,92,93]. Thus, the intervention in this signaling pathway may help alleviate symptoms of related diseases and promote recovery [94].

Zeaxanthin-rich extracts from LB were found to selectively regulate the p38 MAPK pathway in different cells. In the melanoma A375 cell line, zeaxanthin increased p38 expression, resulting in cellular oxidative stress and promoting tumor cell death, which suggests a potential anti-tumor effect [95]. However, in a mouse model of retinitis pigmentosa, zeaxanthin dipalmitate inhibited p38 MAPK activity in retinal tissues, reducing cellular inflammation and oxidative stress-induced cell death. This led to the alleviation and delayed degeneration of retinal photoreceptor structures, meaning that visual acuity was improved [96].

LBPs have been shown to have dual effects on the p38 MAPK pathway depending on the cellular context. In neuronal cells, after brain ischemia, LBPs inhibit the activation of p38 MAPK, preventing inflammatory stress and neuronal cell death [97]. On the other hand, LBPs promote the phosphorylation of p38 MAPK and extracellular-regulated protein kinases (ERKs) in BV-2 microglial cells, increasing reparative autophagy; thus, the cell damage caused by therapeutic micro-electrical pulses is attenuated [98]. This has the potential to reduce the side effects of neurological physical therapy. In addition, LBPs were found to enhance antioxidant capacity through p38 MAPK and peroxisome proliferator-activated receptor gamma (PPARγ) while inhibiting the activities of caspase-3, matrix metalloproteinase-9 (MMP-9), and p53/cyclin-dependent kinase inhibitor 1A (p21). These effects mitigate cellular damage and suppress cellular senescence induced by detrimental environmental factors [99,100,101].

**Figure 6:**
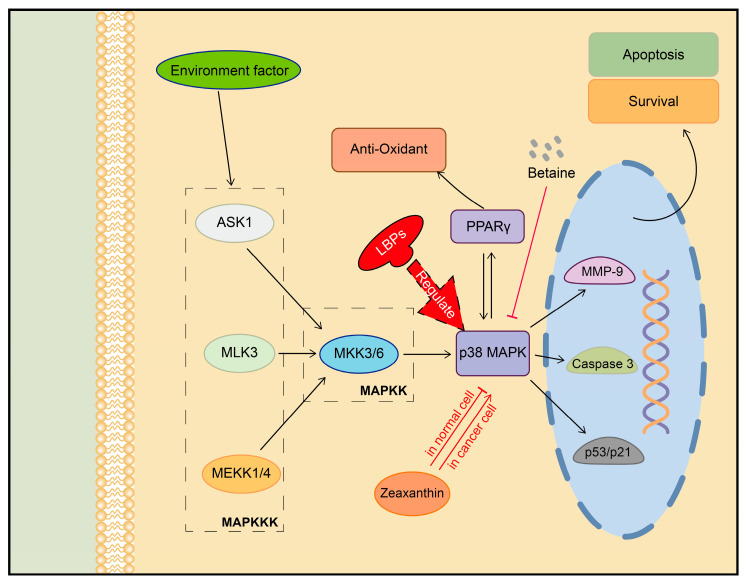
Regulation of p38 MAPK signaling pathway by active ingredients in goji berries. ASK1: apoptosis signal-regulating kinase 1, MLK3: mixed lineage kinase 3, MEKK1/4: mitogen-activated protein kinase kinase kinase 1/4, MKK3/6: mitogen-activated protein kinase kinase 3/6.

Betaine, another bioactive compound found in goji berries inhibits the phosphorylation of p38 MAPK, thereby blocking the chronic inflammation and oxidative stress associated with diabetes. Furthermore, it repairs the damage to the blood–testis barrier and maintains the normal structure and function of the mouse testis [102]. A special LBP named LBP-4a, which can activate both p38 MAPK-α and β, promotes glucose uptake through the activation of glucose transporter type 4 (GLUT4), ameliorating insulin resistance (IR) in Otsuka Long-Evans Tokushima Fatty (OLETF) rat cells [103]. These studies suggest that the active ingredients in goji berries could have therapeutic applications in diabetes-related diseases by targeting the p38-MAPKs signaling pathway (Figure 6).

### 3.6. NRF2/HO-1 Signal Transduction Pathway

NRF2 is a transcription factor that belongs to the Cap’n’collar (CNC)–BZIP family. Under homeostatic conditions, NRF2 is tightly regulated by ubiquitin ligase, formed by Kelch-like ECH-associated protein 1 (Keap1) and Cullin3, meaning that the activated NRF2 is maintained at low levels in the cytoplasm. However, when facing oxidative stress, its degradation is blocked, and the accumulated NRF2 translocates to the nucleus and binds to the AU-rich element (ARE) sequences, initiating the expression of antioxidant-related genes to process the antioxidant response [104,105,106]. HO-1 is a downstream antioxidant enzyme regulated by NRF2 [107,108]. The NRF2/HO-1 pathway is crucial in maintaining systemic redox homeostasis and is strongly implicated in oxidative stress-induced neurological, cardiovascular, and cerebrovascular diseases [109,110,111,112].

Goji berries have been found to improve eyesight. The oral administration of LBPs can significantly increase NRF2 nuclear accumulation and the expression of HO-1 in the retina after acute ischemia–reperfusion injury in rats [113]. Meanwhile, various Chinese medicines containing goji berries have been found to reduce oxidative damage by activating NRF2 and increasing antioxidant enzymes such as HO-1, superoxide dismutase (SOD), and glutathione peroxidase (GSH-Px). These effects have shown promise in the treatment of age-related macular degeneration (AMD) and retinitis pigmentosa [114,115,116,117]. Additionally, LBPs can reduce the oxidative toxicity of H_2_O_2_ in PC-12 cells and suppress CoCl_2_-resulted brain tissue apoptosis in rats, exhibiting protective effects against neurotoxicity by upregulating Nrf2/HO-1 signaling [118]. They also can attenuate the oxidative stress damage induced by light at night in the hippocampus, mitigating cognitive impairment [119]. Lyciumamide A (LyA), isolated from LB, enhances Nrf2 and HO-1 expression and prevents brain ischemia–reperfusion injury (IRI) in the brain [120]. After pretreating the primary cortical neurons of neonate rats with LRPs, the expression of Nrf2/HO-1 was upregulated, leading to a reduction in ROS and cellular damage caused by oxygen glucose deprivation/re-oxygenation. This suggests that LRPs may have potential therapeutic benefits for preventing hypoxic–ischemic encephalopathy (HIE) [121].

**Figure 7:**
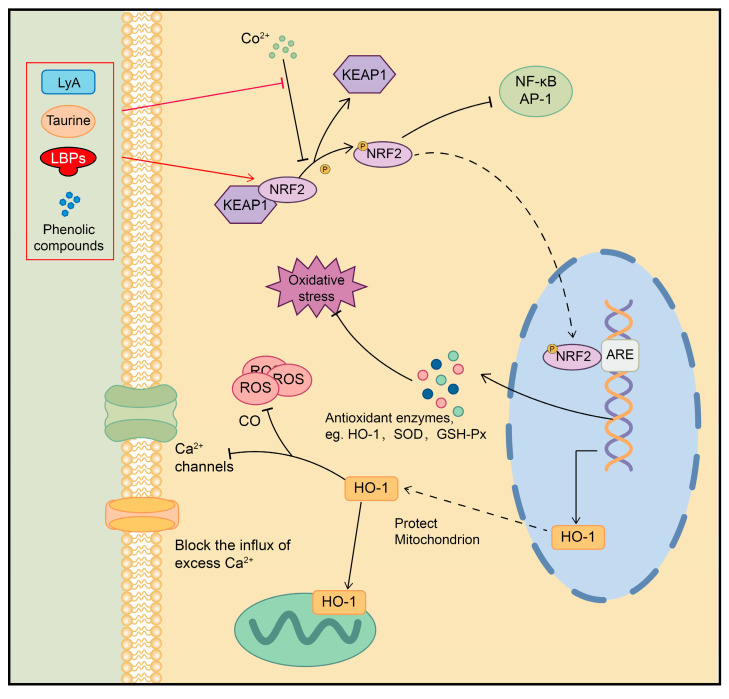
Regulation of NRF2/HO-1 signaling pathway by active ingredients in goji berries. AP-1: Activator protein 1.

In addition, LBPs can improve exercise-induced oxidative stress in muscles and exert anti-fatigue effects [122]. The phenolic compounds in LB enhance the skin’s antioxidant capacity and reduce oxidative stress-induced skin senescence [123]. Taurine (Tau) derived from LB can ameliorate cellular oxidative stress and reduce 5-FU-induced intestinal mucositis in mice through the NRF2/HO-1 pathway [25]. These findings suggest that the modulation of the NRF2/HO-1 signaling pathway could be a critical molecular mechanism underlying the anti-aging and health-keeping effects of goji berries (Figure 7).

### 3.7. NMDAR-Related Signal Transduction Pathway

The NMDA receptor (NMDAR), an ionotropic glutamate receptor, is a critical type of membrane receptor responsible for inter-synaptic signaling. The over-activation of NMDAR can lead to intracellular calcium overload, excessive ROS production, and mitochondrial stress, ultimately resulting in neuron death, which is the primary cause of ischemia-induced nervous system impairment [124]. The NMDAR is mainly composed of NR1 and NR2 subunits [125]. Among these, the NMDAR subtype 2A (NR2A) increases the expression of brain-derived neurotrophic factor (BDNF) by activating the cellular cAMP response element-binding protein (CREB) or AKT pathway, which exhibits neuroprotective effects [126]. However, the activation of another subtype, NR2B (NR2B), leads to severe cellular damage by elevating intracellular ROS levels and inhibiting the CREB pathway [127].

**Figure 8:**
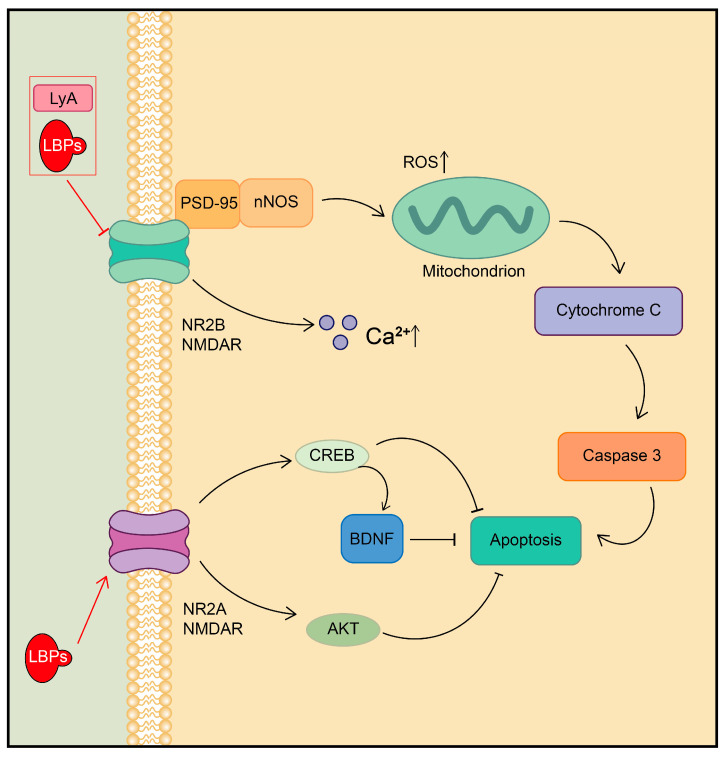
Regulation of NMDAR-related signal transduction pathway by active ingredients in goji berries.

Recent studies have highlighted that LBPs have the potential to protect against mitochondrial damage and apoptosis by inhibiting the formation of the NR2B–postsynaptic density protein 95–neuronal nitric oxide synthase (NR2B-PSD95-nNOS) complex and reducing calcium influx. LBPs were also found to preserve the expression levels of NR2A, pAkt, and pCREB, which are critical for cell survival [128]. LyA, a component derived from LB, inhibits NR2B function by direct binding, thereby preventing Ca^2+^ overload-induced cell death [129]. These findings suggest that the active components in goji berries can regulate the NMDAR-mediated signaling pathway and alleviate neurological damage caused by excitotoxicity (Figure 8).

### 3.8. Regulation of Other Signaling Pathways by Goji Berries’ Active Ingredients

Active ingredients in goji berries can also show health benefits through other cell signaling pathways. For example, LBPs were reported to directly bind to bone morphogenetic protein receptor (BMP) receptors like BMPRIA and BMPRII and propagate signaling through the phosphorylation of the suppressor of mothers against decapentaplegic homolog (SMAD) to improve age-associated bone loss [130]. LBPs increase the expression of stem cell factor (SCF) and its receptor, activating the PI3K pathway to promote testicular cell proliferation and improve sperm quality [131]. Additionally, LBPs have been found to help attenuate inflammatory bowel disease by inducing the conversion of macrophages into anti-inflammatory macrophages (M2-type) through STAT1 and STAT6 pathways [132]. Zeaxanthin dipalmitate from LB acts directly on P2X purinoceptor 7(P2X7) and adiponectin receptor 1 (adipoR1) to restore cellular mitochondrial autophagy, alleviating ethanol-induced liver injury [133]. These results highlight the potential of active ingredients in goji berries to be further exploited for the development of clinically applicable drugs. The discovery and utilization of these compounds could provide new avenues for the treatment of various diseases and conditions.

## 4. Influence of the Active Ingredients in Goji Berries on Gut Microbiota

Microorganisms are ubiquitous in the human living environment, establishing a unique symbiotic relationship with the human host and exerting a significant influence on normal homeostatic balance and pathological disorder [134]. The human gastrointestinal (GI) tract harbors diverse and complex microorganisms called gut microbiota. They possess a huge amount of genetic information, carry out essential metabolic functions, and play a significant role in digestion, metabolism, inflammation, immune function, growth and development, and various physiological processes [135,136]. Numerous diseases, including diabetes, obesity, fatty liver, cancer, and even neurodegenerative diseases, are closely linked to the gut microbiota [137,138,139]. The administration of drugs may disrupt the balance of the gut microbiota, causing unintended consequences that could affect the effectiveness of drug treatments, either generating new health complications or providing new ideas for drug development [140,141]. As a medicinal plant and widely recognized “superfruit” for healthcare, the active ingredients of goji berries closely interact with the gut microbiota and influence the overall state of the body.

**Figure 9:**
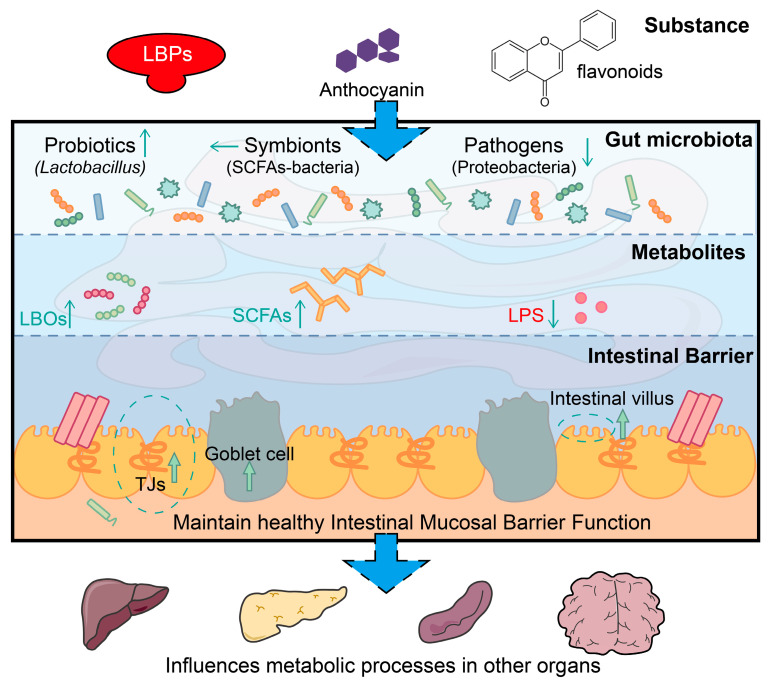
Influence of the active ingredients in goji berries on gut microbiota. LBOs: Lycium barbarum oligosaccharide, LPS: Lipopolysaccharide.

In recent years, there has been a growing body of research on the effects of active ingredients and extracts derived from goji berries on the gut microbiota. Most studies have mainly focused on LBPs, flavonoids, and anthocyanin [142], which can influence microbial species and abundance, as well as their metabolism. The active ingredients also have a positive impact on GI microbial habitats and barrier integrity [143,144]. Anthocyanins can effectively increase the number of intestinal goblet cells and promote mucin synthesis. Tight junction proteins such as zonula occludens-1 (ZO-1), occludin, and claudin-1 are also upregulated to prevent aberrant intrusion across the intestinal barrier [145]. LBPs could enhance the expression of mucin 2 and Claudin5, restore the intestinal barrier, and maintain GI immunity [146]. The active ingredients of goji berries can also improve microbial diversity in the gastrointestinal (GI) tract. For example, they increase the abundance of common probiotic bacteria, including *Bifidobacteria* [147], *Lactobacilli*, and *Lactococcus* [148]; inhibit the growth of disease-related microbes such as *Lachnospiraceae* and *Bacteroides* [149]; and affect microbes associated with the intestinal environment, like *Allobaculum* and *Romboutsia*, which can synthesize short-chain fatty acids (SCFAs) to alter the pH and nutritional status of the gut [148,150,151]. Through their complex influence on GI microbial interactions and interactions between microorganisms and their hosts, as well as the exchange and transformation of metabolites, the active ingredients in goji berries actively participate in immune regulation and energy metabolism and modulate neural messaging [152]. Therefore, they assist in the treatment of various metabolic diseases, including GI tract inflammation [153,154,155], nonalcoholic fatty liver disease [156], alcoholic liver disease [157], and diabetes [149,158,159]. Many studies have shown that LBPs could alleviate cognitive impairment and neuroinflammation caused by high-fat diets [148,160,161]. Recent clinical studies have demonstrated that LBPs can improve symptoms of subthreshold depression in adolescents without adverse side effects [162], indicating the potential of goji berries to be incorporated into daily diets as a healthy food option (Figure 9).

## 5. Perspective on the Active Ingredients of Goji Berries in the Treatment of COVID-19

The SARS-CoV-2 pandemic has severely impacted human society in recent years, causing a series of symptoms known as “long COVID”. This condition has posed a severe challenge for individuals, the healthcare system, and has even impeded the proper functioning of society. Thus, there is an urgent need for effective pharmacological treatments and daily healthcare interventions to alleviate or eliminate the impairment caused by long COVID [163].

A recent study has discovered that LBPs can disrupt the interaction between angiotensin-converting enzyme 2 (ACE2) and viral spike proteins, suppressing viral entry and providing protection against invasion by the Omicron pseudovirus in a K18-hACE2 mouse model, which is a transgenic animal model conditionally expressing human ACE2. This finding showed the possibility that LBPs could act as inhibitors of SARS-CoV-2 viral invasion. The ultimate objective of this research is to develop a nasal mucosal protective agent that can prevent recurrent viral infections, thereby reducing the risk of long-term complications [164].

The mechanisms involved in long COVID encompass various factors and processes, including immune dysregulation, microbiota disruption, clotting and endothelial abnormality, and dysfunctional neurological signaling [165]. Another significant aspect is the presence of post-exertional malaise in long COVID patients, which suggests potential myocyte inflammation, necrosis, and mitochondrial disorders [166]. Several mechanisms contribute to the problems observed in long COVID: 

1. The aberrant activation of multiple signaling pathways, including the NF-κB, Fas/FasL, PI3K-Akt, and p38-MAPK pathways, which promote viral replication, induce pathological inflammatory responses, and organ damage [167,168,169,170,171]. 

2. The prolonged dysregulation of NAD metabolism due to SIRT1 inhibition [172]. 

3. Abnormal antioxidant function resulting from NRF2 inhibition [173]. 

4. Autoimmune disease triggered by the structural similarity between the NMDAR protein domain and viruses [174]. 

5. Viral infections can lead to the abnormal proliferation of intestinal fungi [175]. 

**Figure 10:**
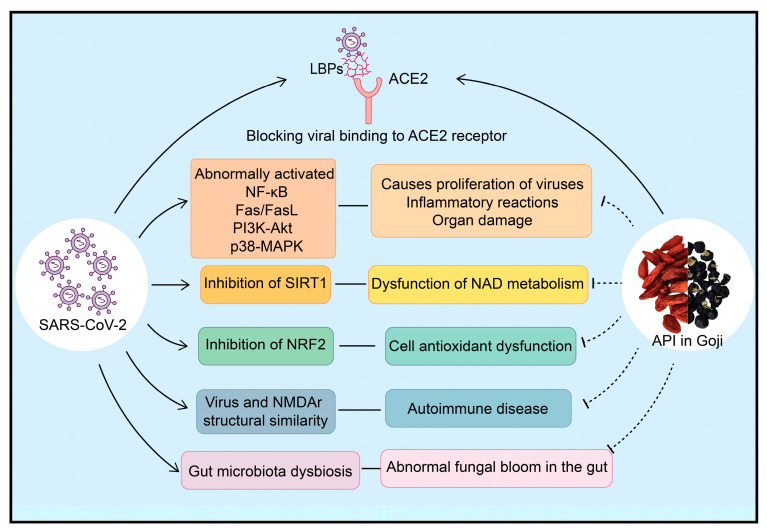
The active ingredients of goji berries may alleviate symptoms caused by SARS-CoV-2.

These mechanisms highlight the complex nature of long COVID and emphasize the need for a multifaceted approach to developing targeted therapies. Certainly, goji berries hold potential as a natural remedy in the management of long COVID symptoms. The active ingredients from goji berries may help improve mitochondrial metabolism, ameliorate inflammation, inhibit abnormal apoptosis, maintain cellular homeostasis, and preserve microbial abundance in the gut through various signaling pathways. These beneficial effects could potentially alleviate and treat the symptoms associated with long COVID. By further exploring and studying the constituents of goji berries, it may be possible to identify and develop effective drugs or therapeutic interventions that can help address the health crisis caused by SARS-CoV-2 and its long-term impacts. However, it is important to note that more research and clinical trials are needed to fully understand the safety and efficacy of goji berries or their derivatives in the context of long COVID (Figure 10).

## 6. Conclusions

Goji berries have gained worldwide recognition for being a highly nutritious food and have a long history of use in traditional East Asian medicine. They are rich in vitamins, dietary fiber, minerals, and other ingredients, making them suitable for culinary and medicinal purposes. Goji berries have a wide range of health benefits, including weight control, keeping the body healthy, and maintaining athletic status [176,177]. This review showed that the active ingredients present in goji berries have the potential to impact multiple cell signaling pathways. It provides a valuable reference for the discovery of drugs targeting different diseases and specific mechanisms. Furthermore, the efficacy of goji berries in treating chronic diseases is often attributed to the interaction of multiple active ingredients and signaling pathways. Therefore, it is important to systematically consider the effect of the active ingredients when studying the therapeutic potential of goji berries and other phytomedicines.

The Lycium genus is widely distributed in many countries, and biogeographic analyses suggest that it originated in South America before spreading to other regions, including Africa, Europe, and Asia [178]. The *Lycium barbarum* L. in goji berries has been cultivated in many countries. In some European countries, several *Lycium barbarum* L. and *Lycium chinense Miller* strains have been selected and cultivated, showing promising market potential [179]. However, only a few strains of Lycium have been effectively utilized, mainly as local ethnomedicines and specialty foods. However, many other strains have not been thoroughly studied or effectively exploited for their potential medicinal value [2,3]. For example, *Lycium Americanum Jacq.* from South America, *Lycium europaeum* L. from the Mediterranean region, *Lycium shawii* from Africa, and *Lycium acutifolium* from South Africa are edible varieties that hold untapped potential for drug discovery and clinical medicine [180]. These species may contain unique chemical constituents and therapeutic properties that could be of interest in future research and development. Further research and investigations are necessary to fully understand the therapeutic properties, safety profiles, and potential applications of these Lycium species in clinical medicine.

## Figures and Tables

**Table 1:** The chemical classes of the major active ingredients and contents of goji berries.

Chemical Class	Main Contents or Compounds (e.g.,)	Major Pharmacological Activity	References
Carbohydrates	Monosaccharides, polysaccharides	Exhibit a wide range of pharmacological activities, including antioxidant, immunomodulatory, anti-tumor activities, and affect gut microbiota	[18,19]
Alkaloids	Tropine alkaloids, pyrrole derivatives, and amide alkaloids (e.g., betaine)	Anti-inflammation, antioxidant	[6,18,20]
Flavonoids	Anthocyanin, ascorbic acid, riboflavin, thiamine, and others	Anti-inflammation, antioxidant, and anti-bacterial	[6,21,22]
Carotenoids	Zeaxanthin, β-Carotene, β-Cryptoxanthin, Mutatoxanthim, lutein, and others	Antioxidant, anti-tumor, and vision protection	[6,18,23]
Amino acid	e.g., Taurine	Anti-inflammation, antioxidant, nutrition, and vision protection	[24,25]
Polyphenols	Lyciumamide A, rutin, caffeic acid, scopoletin, tyramine, and others	Antioxidant, neuroprotection	[26,27]
Others	Lycium barbarum glycopeptides (an immunoactive glycoprotein isolated from the fruit of *Lycium barbarum* L.)	Anti-inflammation, antioxidant, anti-tumor	[28,29]
	*Lycium barbarum* L. seed oil (LBSO; the main contents are fatty acids, especially linoleic acid and linolenic acid)	Antioxidant effect in mitochondria, vascular protection	[6,30]

## Data Availability

All data presented in this study are open-source data, with detailed descriptions given in the cited references.
